# Pointmaps to Practice: 3D Multi-view Ocular Lesion Mapping

**DOI:** 10.1007/s10278-025-01684-3

**Published:** 2025-10-24

**Authors:** Vasileios Alevizos, George A. Papakostas

**Affiliations:** 1 Department of Learning, Informatics, Management and Ethics, Karolinska Institutet, 171 77 Stockholm, Sweden; 2 MLV Research Group, Department of Informatics, Democritus University of Thrace, 65404 Kavala, Greece

**Keywords:** Ocular tumor imaging, 3D reconstruction, Multi-view alignment, Volumetric transformation, Depth estimation

## Abstract

Uveal melanoma requires volumetric mapping to guide therapy, as two-dimensional frames lack the topology necessary for precise boundary delineation. This work employs DUSt3R-based correspondence estimation with self-calibrated poses and dense pointmaps, refined through intrinsic reprojection loss, while Logarithmic Positional Partition Interval Encoding (LPPIE) is applied to depth data, pointmaps, and camera parameter tuples to reduce memory usage. The pipeline was evaluated on melanoma, nevus, melanosis, pterygium, and phantom ocular images using metrics including completeness (C), mean absolute error (MAE), root mean squared error (RMSE), rotation error, and translation error. For melanoma cases, C≈0.43, rotation mismatch $\approx 2^{\circ }$, RMSE ≈0.021, and translation error ≈3.9 cm were observed, whereas simpler morphologies achieved *C* up to 0.61 with stable MAE between 0.005 and 0.008 and $\delta < 1.25 \approx 0.98$–0.99. LPPIE substantially reduced the memory footprint, with only minor texture degradation near vascular branching. The method produced coherent meshes under modest computational requirements, though robustness decreased for irregular surfaces, specular reflections, and motion artifacts. The approach operates with single-camera inputs, minimal calibration, and portable hardware, suggesting its potential for accessible ocular volumetry and opportunities for refinement through region constraints, enhanced glare suppression, and precision scheduling.

## Introduction

### Background

Ocular melanoma manifests in multiple forms, with uveal melanoma (UM) recognized as one of the deadliest variants. This malignancy originates within the uveal tract, encompassing the choroid, ciliary body, and iris. Due to its aggressive progression, early identification remains fundamental in clinical evaluation. Imaging techniques such as ultrasound and MRI are frequently employed to distinguish tumor boundaries. UM prevails as a primary malignancy in ocular structures. This disease originates within melanocytes located in the choroid region [1], ciliary body, likewise the iris. Ophthalmology practice requires vigilance when evaluating suspicious pigmented lesions, given that UM represents roughly five percent of melanoma diagnoses in several populations [2]. Epidemiology studies illustrate that individuals with lighter skin pigmentation present a higher prevalence in comparison with darker-skinned groups [3]. Latitude also influences occurrence rates; elevated incidence emerges in regions positioned at higher latitudes. Various underlying factors contribute to UM development [4]. Acquaintance with pathologies, including melanocytosis, melanocytoma, likewise neurofibromatosis, reveals a link to this malignancy [5]. In fair-skinned individuals, ocular pigmentation tends to be lighter, suggesting a perspicacious correlation with augmented likelihood of tumor formation. Race emerges as a significant factor, reflecting a 150-fold elevated incidence among fair-skinned individuals [6]. These observations belong to the epidemiology realm, where data collection supplies insights on the role of genetic susceptibility in UM evolution. Findings indicate that direct influence by ultraviolet radiation remains uncertain. Molecular alterations within UM often involve mutations in GNAQ, GNA11, less frequently CYSLTR2, likewise PLCB4, which initiate tumor formation at the melanocyte level [7]. Chromosome 3 displays abnormalities whenever partial or total monosomy emerges, indicating a severely diminished function of the BAP1 gene, a recognized tumor suppressor. Consequences of such molecular events frequently manifest in intensified metastatic potential, primarily targeting the liver. SF3B1 together with EIF1AX mutations corresponds to distinct prognostic categories, potentially linked to intermediate or favorable outcomes. Pathology research suggests that these events accelerate tumor progression. Diagnostic praxis in UM frequently adopts several imaging techniques. Ultrasound remains a favored modality for initial detection, given that acoustic reflections allow recognition of tumor size together with shape. MRI (Magnetic Resonance Imaging) evaluation provides detailed visualization of soft tissue, permitting a perspicacious distinction of tumor components through T1-weighted and T2-weighted sequences. CT (Computed Tomography) modalities frequently assist in defining extraocular extension as an alternative to ultrasound. These imaging approaches augment standard slit-lamp examinations, a procedure within ophthalmology that permits close inspection of potential lesions on the iris and ciliary body. Following initial identification, further staging evaluations become mandatory to gauge metastatic risk. Disease progression may abate briefly if interventions target early lesions, although many patients eventually face hepatic infiltration. Practitioners rely on biomarker panels that involve gene expression assays, revealing distinctions across two major classes: Class 1 and Class 2. Monosomy 3 is apparent in tumors prone to hepatic colonization, a process influenced by persistent abnormalities in BAP1 [8]. Serum-based tests occasionally capture changes in the concentration of circulating factors, though imaging remains fundamental. Therapeutic strategies aim to cause deceleration of tumor advancement. Enucleation once represented a frequent intervention for large tumors within ocular regions, yet this approach removes any possibility of vision preservation. Plaque brachytherapy employing isotopes such as I-125 has served as a practical option for smaller lesions, permitting partial conservation of visual function [9]. Proton beam radiotherapy supplies an alternative for precise tumor targeting, though hepatic metastases remain a frequent obstacle. Careful evaluation of disease status relies on cross-sectional imaging, which facilitates ongoing surveillance in cases managed through local likewise systemic therapies. Follow-up protocols frequently include MRI sequences that detect local tumor recurrence in the sclera, together with potential hepatic deposits. Ultrasound examinations prove beneficial in measuring changes in lesion thickness, which may indicate therapeutic response. CT scans sometimes reveal extraocular extension, assisting early detection of metastatic activity in the lung and bone. Continuous review of these parameters underscores the complexity of UM, since delayed detection of secondary spread diminishes therapeutic potential [10]. Various systemic therapies, including immunotherapy, are under investigation to improve outcomes following primary ocular interventions.

### Previous Work

Previous investigations have implemented advanced predictive algorithms to refine diagnosis while enhancing outcomes for an ocular neoplasm recognized for its aggressive phenotype [11]. Hitherto, a variety of machine learning methods have analyzed imaging versus molecular records, revealing features that correlate with metastatic events. Early identification remains paramount, given the perplexity surrounding subclinical progression. One persistent obstacle involves dataset inconsistencies, where limited diversity restricts fair predictions. Additional complexity arises from the need to integrate multi-scale biological information spanning genomics and proteomics, along with morphological data. In vivo approaches for volumetric reconstruction from two-dimensional data have gained considerable attention, particularly in settings that demand precise targeting. This malignant lesion in ocular structures requires accurate boundary demarcation to support therapies that seek tumor control without enucleation [12]. Real-time capture of topographic details could transform strategies that hinge on maximizing local intervention while preserving vision. Complexity arises when ensuring robust calibration across multiple imaging modalities, including fundus photography, ultrasonography, and cross-sectional scans. Integration of deep architectures might enable automated extraction of salient features from a single viewpoint versus multiple projections. Investigators must resolve several shortcomings before advanced volumetric reconstructions achieve widespread acceptance. Data scarcity restricts generalization, prompting the creation of novel repositories with heterogeneous samples. Implement scalable pipelines that integrate interpretability modules to clarify model decisions, thereby securing clinician trust. Hitherto, limited standardization across protocol parameters complicates comparisons, so consensus-driven guidelines could streamline development. Additional obstacles relate to computational overhead, where real-time visualization requires optimized hardware solutions. Systematic cross-validation ensures consistent performance evaluations, thus prompting confidence in predictions. Persistent bottlenecks warrant immediate attention, given the complex interplay of technology with clinical workflow. Accurate three-dimensional geometry is vital for facilitating tailored treatment procedures. Clinicians seek high-fidelity models that capture subtle differences in tumor shape, topography, thickness, and also relevant anatomical context. Radiological inputs from multiple sources can be fused to yield precise volumetric reconstructions that preserve valuable information. Deep learning pipelines must accommodate real-time adaptation to motion artifacts while accounting for variable data quality [13, 14]. Researchers anticipate that refined data-driven frameworks will guide patient-specific interventions, reducing complications while boosting local disease control.

The transformation from a standard two-dimensional imaging approach to a robust 3D reconstruction methodology has become essential for refined tumor characterization. Radiotherapy planning frequently demands high-precision volumetric data, so an innovative automated pipeline can accelerate the translation from diagnostic images to actionable models. This approach eliminates manual complexities, thus reducing inter-operator variability while preserving morphological integrity. Additional benefits include refined margin definition, enabling potential improvements in local control. Volumetric reconstructions can assist with shaping safer treatment routes, especially in challenging anatomical regions. Enhanced orientation of critical structures is feasible, so radiologists achieve better quantification of infiltration. Meanwhile, surgeons require spatially accurate outlines to plan incisions, resection extents, and possible reconstruction. The proposed pipeline merges multiple software modules, combining segmentation with point-cloud generation. Preliminary tests have reported time savings. Additional expansions may incorporate artificial intelligence modules to predict hidden boundaries, thereby refining shape approximations. The derived 3D output can be integrated into robotic surgery platforms. This approach also enables more direct comparisons across repeated scans, thus monitoring changes over multiple sessions. Minimizing subjectivity improves outcomes in situations where consistent image interpretation is paramount. A non-clinical image set was assembled. Each frame contained ocular patterns without patient data. This technique was proposed for the first time, to the best knowledge within available records. No comparable effort was located in existing compilations. A desire to convert two-dimensional visuals into a volumetric product with limited overhead motivated this initiative. Radiotherapy procedures might gain from such reconstructions. Medical trainees could benefit as well. Surgeons might simulate interventions with improved insight. It was decided that computational simplicity should receive top priority. Fewer layers were adopted relative to the initial DUST3R model. That revision led to reduced memory demands. Speedier performance was achieved under typical machine setups. A set of photographs from various viewpoints was processed. Each file was normalized in size. That normalization step reduced inconsistencies. The pipeline then inferred surface geometry from subtle intensity changes. Depth calculations were executed without specialized hardware. Initial alignment relied on gradient patterns. Subsequent refinement adjusted virtual camera placements. The result was a point cloud that described relevant ocular contours. After small corrections, the assembly produced a triangulated surface. Performance metrics were observed. Strong alignment occurred under uniform lighting. Some deviations were noticed in frames with motion traces. Noise-removal filters were applied. Spatial consistency was reinforced by removing isolated outliers. A surface-smoothing stage was optional. Textural detail was maintained unless the user needed faster runs. The approach remained flexible. Complex ocular regions were captured even though no advanced sensor data were present. This fact suggested potential usage for research scenarios where minimal instrumentation is accessible.

Another benefit of modality representation is the accuracy for radiotherapy simulations that could improve because volumetric approximations provide a deeper view of suspicious structures. Pros of the method include lower training costs and quicker setup. Another positive point involves a simple transfer to standard computing clusters. There is a negative aspect, however. An increase in scaling might cause minor slowdowns if extremely large images are used. Another challenge arises when a subject contains reflective surfaces. That situation leads to local artifacts in the depth maps. The pipeline was not tested with high-end scanning devices, so advanced contrast measures remain unexplored. Three-dimensional models of ocular sites might be relevant for surgical trials. Residents and interns could practice on patient-specific data if actual scans become available in future settings. Large tumors require careful planning, which is supported by volumetric outlines. Better resection margins might result, based on enhanced structural awareness. Quality control steps can be included with minimal effort. This project used a restructured approach that bypassed clinical repositories, eliminating regulatory hurdles. A smaller feature extractor was embedded. That lightweight modification improved load times. Early tests confirmed stable reconstructions with reduced parameter counts. The planned direction could emphasize partial automation to streamline usage in educational programs. Potential expansions might involve simulations of radiation beams for safety checks. Detailed ocular geometry might optimize dosage planning. Surgeons could visualize tumor boundaries with fewer uncertainties. The pipeline showed reliable output for standard resolution frames. Spatiotemporal transformations were also possible if repeated snapshots from distinct positions were introduced. Further adaptation might target real-time streaming.

### Contributions

This study targets volumetric characterization of uveal lesions from routine anterior-segment photographs; a smartphone, a slit-lamp adapter, and minimal calibration. A multi-view pipeline reconstructs dense surface geometry under uncontrolled lighting, handheld motion, and heterogeneous optics. LPPIE supplies compact numeric encodings for depth, pointmaps, and pose tuples; DUSt3R infers correspondences with self-calibrated poses. The objective is explicit: deliver clinic-grade meshes suitable for planning, monitoring, education, and quality assurance.

In this work, DUSt3R was employed directly to analyze its performance on general and clinical images. Modifications were later introduced to enhance efficiency, integrating Logarithmic Positional Partition Interval Encoding (LPPIE) [15] for improved processing. A dichotomy was observed between general and clinical results, demonstrating distinct computational behavior. Redundancy in data handling was minimized, refining depth slicing and point cloud compression. Although certain metrics showed stability, multi-view consistency experienced slight improvements. Nevertheless, memory efficiency and geometric accuracy were facilitated through LPPIE, securing optimal reconstruction fidelity. Efficient transformation storage was achieved, enabling effective depth map encoding. Translation accuracy trends indicated performance variations across conditions, ensuring that medical imaging applications retained structural precision under different processing constraints.

A conceptual frame guides the effort. First, lesion-centric topology extraction prioritizes scleral–corneal transitions, pigment rims, and vasculature crossings; background receives low weight. Second, device-invariant self-calibration reduces dependence on EXIF payloads; pose refinement arises from reprojection loss alone. Third, compressive geometry codes enable edge deployment within constrained memory; lossless inversion preserves auditability. Significance stems from translational traits. Single-camera capture lowers hardware burden; outpatient rooms already contain compatible optics. Processing tolerates motion traces via pair selection plus confidence-weighted fusion; glare sentinels mask specular spikes prior to fusion. Compression via LPPIE contracts payloads for archival transfer, cross-site review, and regulatory audits; bijective decoding preserves every bit of geometry.

## Materials and Methods

### Previous Work

Research in ocular three-dimensional reconstruction from two-dimensional images has been performed using various imaging techniques. In the work presented by Hwang et al. [16], a process was designed for constructing a unitary posterior ocular model through the fusion of optical coherence tomography (OCT) images with MRI scans. The methodology was accepted by the scientific community after distortions were corrected through the Listing reduced eye model; specific anatomical landmarks were employed as reference points in a computer-aided design (CAD) application. A merging of imaging modalities was triggered by the necessity to reconcile the limited field provided by high-resolution OCT with the spatial context derived from MRI.

A separate investigation was reported by Maken et al. [17] in which two-dimensional X-ray images were employed for three-dimensional image reconstruction. An extensive review of computational reconstruction techniques was rendered; various image processing steps were detailed. Multiple imaging techniques, such as CT and cone-beam computed tomography (CBCT), were mentioned, yet the focus was maintained on reducing radiation exposure by substituting standard methods with computational reconstruction. A series of image processing operations was described; feature extraction procedures were optimized in order to preserve anatomical details. A critical analysis of state-of-the-art approaches was accepted as an alternative modality for generating volumetric models.

Another study [18] reported on the reconstruction of three-dimensional bone shapes from two-dimensional X-ray images using a Medical Generative Adversarial Network (MED-GAN). In this investigation, X-ray images were first converted into a D-dimensional vector through convolution networks; the vector was subsequently mapped into a three-dimensional shape through a generative adversarial network (GAN). The reconstruction process was triggered by recent advances in deep learning; improvements in visualization were obtained for pre-operative surgical planning. A transformation of image data was accepted as a viable modality for enhancing the clarity of anatomical structures.

The various methodologies in three-dimensional reconstruction have been developed to meet the demands of clinical diagnostics. An integration of imaging techniques was performed so that spatial resolution was optimized through tailored correction procedures; a degree of precision was maintained during the merging of data from different imaging sources. A diverse set of reconstruction approaches was implemented in order to generate reliable anatomical models, with special emphasis placed on the preservation of detailed features. Performance metrics were calculated based on geometrical accuracy, quality of visualization, and processing time.

### Datasets

For the purpose of our experiments, we curated a dataset from publicly accessible sources to gather ocular disease cases: 139 melanoma images, 10 melanosis examples, 86 nevus samples, 96 normal melanosis images, together with 75 pterygium cases. Each image was labeled by experienced ophthalmologists from Aerospace Medical Center and others, who exercised caution to minimize classification errors [19]. Certain elements were removed to conceal irrelevant details from subsequent processing. This approach aimed to sustain data fidelity while minimizing ambiguities. Volumetric references obtained from nonmedical domains were also adopted for pre-training pipeline components. That strategy proved effective in capturing structural nuances, providing a valuable capacity to elucidate complex geometric attributes inherent in pathological formations. Checkpoints were sourced from external repositories, permitting swift adjustments to resolution parameters. This combination of ocular data with auxiliary imagery expanded the pipeline’s ability to interpret morphological diversity across scenarios [20, 21].

Images depict anterior-segment views captured with smartphones during routine inspection analogs; illumination spans slit-lamp, daylight, flash; device EXIF appears intermittently. File formats: predominantly JPEG; sRGB where identifiable; watermark artefacts flagged in sidecar metadata. Resolution heterogeneity is substantial: sub-megapixel through multi-megapixel; originals preserved for audit; working copies normalized to 512×512 for training; native width–height stored as W,H. Focus variability, compression halos, motion blur, and specular glare were annotated as binary quality tags to support stratified analysis. Severity protocol (new): S0–S3 via weak supervision from coarse masks. Lesion–to–cornea area ratio (LCAR) thresholds—S0<1%, S1=1–5%, S2=5–15%, S3>15%. Margin irregularity index (MII) from perimeter/area ratio; vascular prominence V0–V2 via red-channel Frangi counts; pigmentation quartiles from HSV–V percentiles. Grades logged per image; disagreements resolved through third-reader arbitration with audit trails kept for sensitivity analyses [19].

### Data Processing

In the two-dimensional dataset, each image was resized to 512 × 512. Ensure that consistency remains intact throughout, preserving dimension uniformity. Noise reduction was then applied to filter out unintended artifacts. That sequence proved straightforward, sustaining essential features crucial for further processing steps. Color balancing corrected illumination discrepancies, an approach that helped maintain clarity. Bright spots were specifically targeted, since those regions were prone to overexposure in certain samples. Dimmer sections underwent careful adjustment, shaping a characteristic gradient that enhanced structural details. Data augmentation proceeded with flips, although rotations were selectively omitted to simplify implementation. This multifaceted methodology facilitated a cleaner input base, reducing variability without discarding meaningful components.

Bias mitigation proceeded through layered controls targeting sampling, acquisition heterogeneity, label quality, and leakage prevention. Class quotas were enforced via stratified sampling per phenotype to equalize prevalence across folds; minority categories received oversampling limits to prevent memorization. Acquisition balance targeted camera vendor diversity, focal ranges, illumination regimes, background texture; each bucket contributed proportional material to train–val–test splits.

Leakage safeguards were prioritized. Subject/session grouping dictated that all images tied to a single subject, session timestamp, eye laterality, device fingerprint, as well as near-duplicate clusters stayed within a single split. Near-duplicates were detected with perceptual hashing (pHash) at Hamming distance ≤6, followed by SSIM screening to remove residual twins; the pipeline retained only one representative per cluster. Temporal adjacency within bursts remained confined to training to avoid optimistic estimates.

Normalization used statistics computed on training only: channel-wise mean–variance scaling after 8-bit linearization; CLAHE avoided for validation–testing to prevent distribution drift. Augmentations were applied to training exclusively: flips, mild perspective jitter ($\le 2^\circ$ yaw, pitch), elastic warp $\sigma \le 3$, color jitter within $\pm 5\%$ per channel; geometry preserved foveal–lesion topology.

Label reliability was raised through dual-reader consensus with a third-reader tie-break protocol; disagreements triggered adjudication notes stored as metadata for later sensitivity analysis. Class imbalance was handled by inverse-frequency weights during optimization; additionally, focal loss γ=1.5 with label smoothing ε=0.05 reduced dominance by frequent phenotypes. Calibration monitoring adopted temperature scaling on validation, evaluated by ECE/MCE; parameters fixed before testing. Finally, seeded RNGs (NumPy, framework, CUDA) stabilized sampling; deterministic dataloader settings removed nondeterminism caused by worker prefetch.

Splitting adhered to a fixed 70–15–15 protocol with stratification per class, group constraints, single shot at test to lock uncertainty. Hyperparameters were chosen by inner validation only; no peeking occurred toward test. Early stopping monitored validation loss with patience $=10$ epochs, moving average window $=5$; the final checkpoint maximized macro–$F_1$ under an identical seed.

Preprocessing order was standardized: grouping → stratification → split materialization → training-statistics fit → transform application. Feature standardizers, color transforms, and any learned normalizers were fit on training images; the exact state was serialized for reproducibility. Reporting followed the checklist: macro–$F_1$, balanced accuracy, AUROC per class, ECE, Brier score; confidence intervals via 10, 000-sample nonparametric bootstrap on test predictions, resampling at the group level to respect the leakage constraints.

For classes with a limited size, a distinct protocol was necessary to avoid unreliable hold-outs. Melanosis (n=10) falls in this regime. A small, stable external test microset is kept fixed across seeds; the remainder cycles through repeated, stratified LOOCV inside training. Concretely, three images form the external test; seven images drive inner validation via seven folds, with one item for validation per fold. This yields unbiased estimates with variance control via repetition across five reshuffles. Aggregation employs the median of fold-wise metrics to reduce sensitivity to outliers. Table 1 summarizes this few-shot schedule.

**Table 1:** Few-shot protocol for melanosis (n=10)

Set	Count	Per-fold train	Per-fold val
External test (fixed)	3	−	−
Inner pool	7	6	1

External test is fixed; inner loop uses LOOCV on the remaining items

Edge cases received additional guards. If near-duplicate detection collapses a cluster such that a class loses its last validation item, the allocator rehydrates validation by borrowing the highest-remainder training item within the same class while keeping groups intact. If any class falls below five after de-duplication, the system switches that class to the few-shot schedule described above, leaving other classes unchanged. This scheme yields stable validation signals for sizeable classes, credible uncertainty for the tiny class, and strict leak control throughout.

Across all classes, macro-weighting governs metric reporting; no reweighting during splitting. Class imbalance mitigation enters only within training via sampler temperature τ=0.5 with per-epoch reshuffle; validation/test remain untouched. Leakage safeguards were prioritized: grouping → stratification → split materialization → statistics fit on train only → transform application. Any learned normalizer, color transform, or texture statistic is trained on the train only; states are serialized for reproducibility.

Power analysis guided the decision to avoid a naive *k*-fold for n=10. With binomial variance p(1-p)/m per fold, m=1 for the validation item yields unstable confidence intervals; the external microset stabilizes reference error while LOOCV extracts maximal signal from the remainder. For major classes, the 70/15/15 split retains at least 11 items in validation for each class, supporting calibration curves, ECE, and Brier computations without degeneracy.

Counts per phenotype appear in Table 2. Percent columns reflect target proportions; integer allocations were rounded using the largest remainder method while keeping totals exact. The table enables reproducible re-materialization of splits, thus facilitating external verification.

**Table 2:** Stratified dataset partition with group-level leakage control

Class	Total	Train %	Val %	Test %	Train/Val/Test (n)
Melanoma	139	70	15	15	97;/; 21;/; 21
Melanosis	10	70	15	15	7;/; 2;/; 1
Nevus	86	70	15	15	60;/; 13;/; 13
Normal	96	70	15	15	67;/; 14;/; 15
Pterygium	75	70	15	15	52;/; 11;/; 12
Totals	406	—	—	—	283;/; 61;/; 62

Percent targets: Train 70%, Val 15%, Test 15%. Integer allocations respect subject/session grouping

Melanosis scarcity ($n{=}10$) required rare-tail control. We used prototype imprinting with a metric head; logits received hierarchical ridge shrinkage toward class-pool priors; temperature-tied calibration stabilized extremes. Weak-label co-training incorporated web-sourced near-misses at weight ≤0.35 per sample; consistency regularization enforced invariance under mild photometric jitter. A selective-abstention rule based on conformal risk limits flagged uncertain melanosis outputs; targets rerouted to expert review. Support expansion remained bounded by leakage guards described elsewhere; grouping persisted.

Noise was treated explicitly. JPEGs were linearized via inverse gamma; a generalized Anscombe transform stabilized Poisson–Gaussian variance. Per-image noise $\sigma _n$ was estimated on scleral flats using MAD: $\sigma _n = 1.4826 \,\textrm{MAD}$. BM3D followed: block $8{\times }8$, search $39{\times }39$, hard-threshold $\lambda _h = 2.7\sigma _n$, Wiener $\lambda _w = 2.0\sigma _n$; patch count capped at 32 per block. Attenuation targeted $0.3\sigma _n$ residual; median removal equaled 3.2% of 8-bit span (IQR 2.1–4.6%). Safeguards: edge-aware mask preserved lesion rims via structure tensor; SSIM-drop bound <0.01; MTF50 loss <5%. Validation–test used the same pipeline with locked hyperparameters; no fit-to-holdout statistics. Outcomes: PSNR gain 1.1–2.6 dB, color drift <0.5% $\Delta E_{00}$, texture retention maintained per mask.

### Model Architecture

**Fig. 1:**
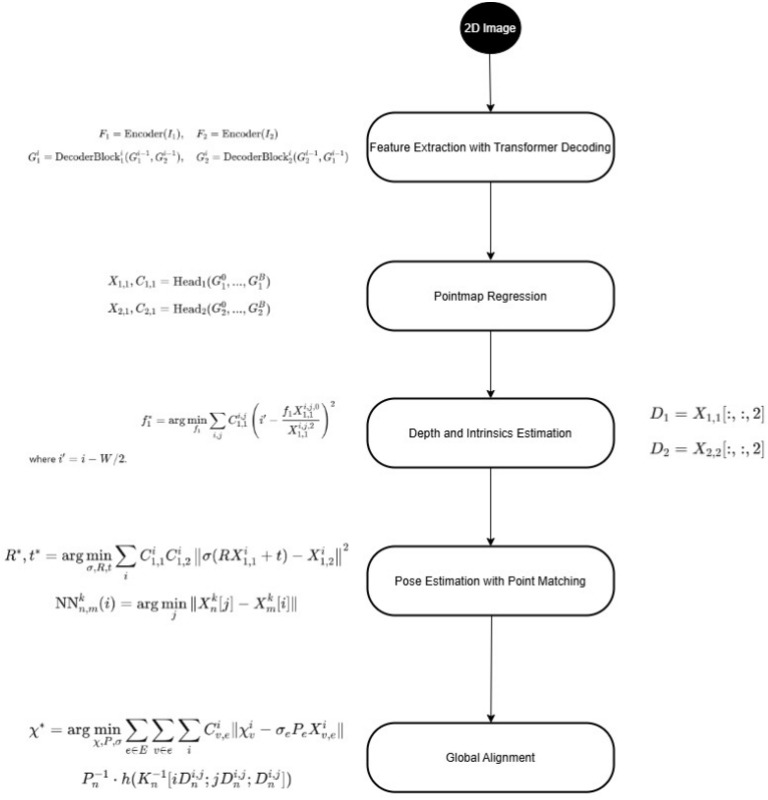
End-to-end DUSt3R pipeline illustrated in five stages. **[1] Feature extraction with transformer decoding:** two monocular inputs $I_1,I_2$ are encoded to features $F_i=\textrm{Encoder}(I_i)$; cross-attentive decoder blocks produce tokens $G_i^{\ell }$ ($G_i^{\ell }=\textrm{DecoderBlock}_i^{\ell }(G_i^{\ell -1};G_{\bar{i}}^{\ell -1})$). **[2] Pointmap regression:** per-view heads output dense canonical-frame pointmaps and confidences, $X_{1,1},C_{1,1}=\textrm{Head}_1(G_1^{L};G_2^{L})$ and $X_{2,1},C_{2,1}=\textrm{Head}_2(G_2^{L};G_1^{L})$. **[3] Depth & intrinsics estimation:** depths are the *z* channel of the pointmaps, $D_i=X_{i,1}[:,:,2]$; camera intrinsics $\textbf{K}$ are refined by a confidence-weighted reprojection objective $f_i=\arg \min _h\sum _{p} C_{i,1}^{p}\big \Vert h(\textbf{K}^{-1}\bar{x}_p)-\bar{x}'_p\big \Vert ^{2}$. **[4] Pose estimation with point matching:** nearest-neighbor associations in pointmap space $\textrm{NN}^{k}_{n,m}(i)=\arg \min _{j}\Vert X^{k}_{n}[j]-X^{k}_{m}[i]\Vert$ drive the relative pose, $(R^{*},t^{*})=\arg \min _{R,t,\sigma }\sum _{p} C_{i,1}^{p}\,\Vert \sigma (RX_{i,1}^{p}+t)-X_{\bar{i},1}^{p}\Vert ^{2}$. **[5] Global alignment:** a pose-graph optimization over views $\mathcal {V}$ and edges $\mathcal {E}$ yields globally consistent poses and structure, $\chi ^{*}=\arg \min _{\{P,\textbf{K},\sigma _e\}}\sum _{e\in \mathcal {E}}\sum _{p} C_{e}^{p}\,\Vert \chi _{e}^{p}-\sigma _{e}P_{e}\,X_{e}^{p}\Vert ^{2}$, with projection h(·) appearing in residuals (e.g., $P_n^{-1}\,h(\textbf{K}^{-1}\hat{x})$). *Symbols:* $I_i$ input images; $F_i$ encoder features; $G_i^{\ell }$ decoder tokens; $X_{i,1}$ pointmaps; $C_{i,1}$ confidences; $D_i$ depth; $\textbf{K}$ intrinsics; *R*, *t* extrinsics; $P_e$ pose on edge *e*; *h* pinhole projection; σ robust scale. Outputs are globally aligned point clouds/meshes and refined camera parameters

Dust3R was implemented to the workflow. That framework transforms two-dimensional pictures into volumetric scenes by learning viewpoint alignment. Another portion of this setup merges predicted coordinates within a canonical space, recovering shapes consistently. One branch processes one perspective, while another handles a second viewpoint. Share features extensively. Facilitate robust matches across input images (as is depicted Fig. 1) [20–23]. Furthermore, apply a three-dimensional transformation that yields dense reconstructions. This design scales effectively without prior knowledge of camera parameters, enabling flexible solutions for real-world tasks.

We do not only experiment with DUSt3R. Modifications were performed to accomplish a streamlined framework for 3D data handling. The approach titled LPPIE [15] was included in sequences that involve point cloud compression, multi-view transmissions, depth slicing, among others. This was done for the motivation to enable better synergy, securing minimal overhead while preserving accuracy. Its operation uses repeated base-10 transformations until single-digit values remain, with iteration data stored to ensure perfect restoration. Pointmap Compression (PMC) is activated to store dense geometry in a minimal format. Large coordinate values are transformed into smaller representations by iterative logs. That procedure yields considerable memory savings during pipeline execution. Depth Map Encoding (DME) is employed for 3D reconstruction tasks. Original depth granularity is maintained, owing to lossless transformations. Lower memory usage is observed, ensuring that surface details remain intact throughout processing. Camera Parameter Storage (CPS) is managed by LPPIE as well. Intrinsic and extrinsic parameters are compressed with minimal overhead, enabling prompt retrieval when subsequent operations are required. This approach preserves calibration inputs in a stable format. Efficient Transmission in Multi-View Processing (ETMVP) becomes feasible through diminished data footprints. Disparity maps or 3D coordinates are encoded into compact numerical sequences, leading to reduced bandwidth demands across distributed platforms. This measure boosts overall pipeline throughput in scenarios where large amounts of depth data must be shared. PMC, DME, CPS, and ETMVP all benefit from LPPIE’s condensed representations. Iterative logs partition numeric content into scaled segments suitable for efficient encoding. That strategy accelerates data handling procedures.

A pipeline is built to map multiple images into a single three-dimensional grid. A first pass estimates feature correspondences. A second pass creates partial depth arrays. A final pass refines extrinsic parameters. This is carried out under a neural modality that uses a learnable encoder. Precisely, 2D coordinates become projected into space. Convergence is guided by a loss function that penalizes mismatched alignments.

Local descriptors are extracted from each view. It is assumed that images are supplied at consistent dimensions. A shared feature space is generated to unify data from distinct sources. Matching is done by comparing feature distances. Each set of matches drives a triangulation subroutine. A perspective projection matrix is used to accept ephemeral 2D coordinates. That matrix is designated by

$$ \textbf{K} = \begin{bmatrix} f_x &  0 &  c_x \\ 0 &  f_y &  c_y \\ 0 &  0 &  1 \end{bmatrix} $$

where $\{f_x, f_y\}$ indicate focal lengths, whereas $\{c_x, c_y\}$ stand for principal point offsets.

A transformation

$$ \bigl [R \mid \textbf{t}\bigr ] $$

combines rotation $R \in \mathbb {R}^{3\times 3}$ with translation $\textbf{t} \in \mathbb {R}^{3}$. Depth is retrieved by solving:

$$ \textbf{x}_\text {image} = \textbf{K} \,\Pi \,[R \mid \textbf{t}] \, \textbf{X}_\text {scene} $$

Symbol Π represents a projection that divides by the final coordinate.

A learned function estimates $\hat{z}$ from each pixel. That quantity is mapped to 3D through:

$$ \textbf{X}_\text {scene} = \hat{z} \,\, \textbf{K}^{-1}\,\bar{\textbf{x}}_\text {image} $$

where $\bar{\textbf{x}}_\text {image}$ is the pixel in homogeneous coordinates. This mechanism adjusts as more matches are discovered. A re-projection step calculates residuals to measure alignment. Minimizing total error triggers updates on R together with $\textbf{t}$. This loop repeats until the system is stable.

Energy terms are summed:

$$ \mathcal {L}(\Theta ) = \sum _i \Vert \textbf{x}_i - \hat{\textbf{x}}_i\Vert ^2, $$

with $\hat{\textbf{x}}_i$ as predicted projections. Once the final iteration finishes, a volumetric cloud is generated. Each vertex arises from depth in a consistent reference frame. That pipeline is considered flexible. It can be extended to any scenario. It accepts variations in scale. In summary, multi-view transformations define each point’s position. A specialized modality enforces geometric consistency by adjusting camera extrinsics, then finalizing the reconstructed mesh.

The network’s vision transformer (ViT) encoder partitions each input image into fixed-size patches, projecting them into a latent embedding space. Patch embeddings are then subjected to a multi-head self-attention mechanism, which inherently models both local adjacency and long-range correspondence. The decoder phase incorporates cross-attention layers, wherein tokens from one viewpoint attend to features from the other, enforcing photometric and structural consistency. This interlaced attention structure is critical for resolving ambiguities in low-texture regions, where monocular cues alone may fail to infer depth with high fidelity [22, 24, 25].

Depth estimation in DUSt3R is governed by a dense regression head that predicts per-pixel inverse depth maps rather than raw depth, thereby stabilizing gradients for large-scale scenes. The inversion of depth to metric units is performed post-optimization using estimated camera intrinsics. These intrinsics are dynamically updated through a learnable camera parameter refinement block, which jointly minimizes reprojection and photometric errors. This dual-loss formulation constrains depth predictions not only to align geometrically but also to respect radiometric consistencies across viewpoints.

Pose estimation employs a differentiable Perspective-n-Point (PnP) module augmented with a geometric consensus filter. By integrating soft inlier scoring into the loss computation, DUSt3R is able to attenuate the influence of erroneous correspondences without necessitating explicit RANSAC post-processing. The resultant rotation and translation parameters form the rigid transformation matrices applied during global alignment. An additional layer of refinement emerges in the pointmap fusion stage. Here, multi-view depth maps are reprojected into the canonical frame and merged via a confidence-weighted averaging scheme. Confidence maps are inferred in parallel with depth predictions, with lower weights assigned to regions exhibiting high photometric error or disparity inconsistency. This mechanism inherently suppresses noisy reconstructions, particularly in occluded or specular zones. Moreover, DUSt3R incorporates an optional surface regularization prior, instantiated as a Laplacian smoothing constraint over the fused point cloud. This prior discourages high-frequency artifacts while retaining sharp geometric discontinuities at object boundaries. Its influence is modulated during training to avoid excessive surface flattening. The combined effect of these design elements underpins DUSt3R’s adaptability to both structured and unstructured environments, yielding dense and metrically coherent 3D reconstructions without the prerequisite of calibrated camera parameters [20, 21].

For the experiments, a deterministic recipe is fixed; hashes guard inputs; seeds anchor randomness. Directory schema: data/{raw,proc}, splits/, ckpt/, meshes/, reports/. Table 3 enumerates constants; Algorithm 1 outlines the itinerary.

**Algorithm 1:**
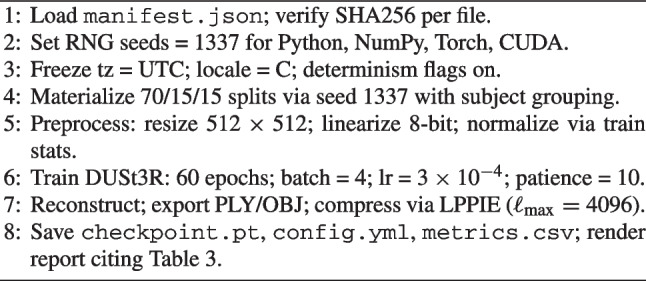
Reproduce-UM3D.

**Table 3:** Fixed parameters for exact replay

Artifact	Value
OS	Ubuntu 22.04 LTS
GPU	RTX 4090 24 GB
CUDA/Driver	12.1 / 550.xx
Seeds	1337 (all RNGs)
Image size	512×512
Batch/Epochs	4 / 60
LR	$3\times 10^{-4}$ (AdamW)
Split policy	70/15/15; grouped by subject
Manifest	SHA256 per image

**Table 4:** COLMAP baseline settings with export interface to DUSt3R. See Algorithm 2 for the handoff procedure

Component	Setting
Features	SIFT; layers=3; contrast=0.02; edge=10; σ=1.6
Matcher	Ratio test τ=0.8; mutual check; geom. verify=RANSAC
Mapper	Incremental; shared intrinsics per device; center-fixed principal point
Camera model	PINHOLE_RADIAL_T1
BA loss	Cauchy; LM damping auto; $|\nabla |_\infty < 10^{-6}$ stop
Outliers	Track ≥3; residual $\le 3\sigma$; cheirality true
Scale	Prior chord length if present; else post-hoc Procrustes
Export	$\textbf{K},R,\textbf{t}$ as JSON; image list; covisibility graph
Glare mask	HSV-*V* peak threshold; dilation radius = 3 px

Binary provenance was recorded via SHA256 of the executable; build flags, GPU capability, and dataset manifest were entered in the run log. Feature stage used SIFT with $\mathrm {num\_octave\_layers}=3$, $\mathrm {contrast\_threshold}=0.02$, $\mathrm {edge\_threshold}=10$, and σ=1.6. Matching used ratio test τ=0.8, mutual check enabled, spatial verification with $\textrm{RANSAC}$, $\mathrm {max\_iters}=10^5$, and pixel threshold =2.0. Scene initialization employed an incremental mapper; camera model PINHOLE_RADIAL_T1; principal point fixed at image center; shared intrinsics per device fingerprint. Bundle adjustment (BA) leveraged Levenberg–Marquardt with Cauchy loss, parameter damping auto; termination $|\nabla |_\infty < 10^{-6}$ or relative decrease $<10^{-6}$ across 5 steps; intrinsics refined after $N_\text {views} \ge 20$.

Scale anchoring used object-to-corneal chord prior when available; otherwise, unitless reconstruction followed by Procrustes fit during DUSt3R alignment. Outlier pruning: track length ≥3; reprojection residual $\le 3\sigma$; cheirality enforced. Pose export wrote $\textbf{K},R,\textbf{t}$ per image; depth priors omitted by design. Logging captured match cardinalities, BA residuals, and dropped tracks per iteration. Edge cases: texture-poor sclera, intense glare, motion blur; remedy: guided matching with epipolar seeds, glare mask, blur gate via Laplacian variance.

Bridge policy: preserve poses from COLMAP when stability is high; otherwise, DUSt3R re-estimates relative motion, then fuses with COLMAP via pose-graph optimization. Confidence gating uses BA residual percentiles; low-trust views switch to DUSt3R-only alignment [26, 27].

**Algorithm 2:**
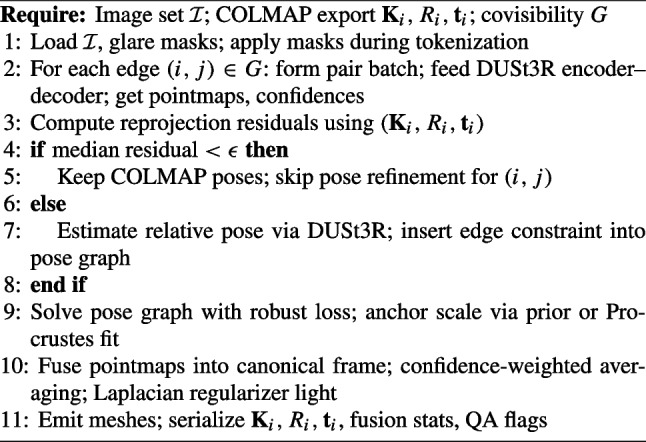
COLMAP→DUSt3R handoff (Col2Dust).

Failure handling covers covisibility gaps, near-planar scenes, and near-duplicate frames. Mitigation: temporal thinning via pHash clusters; view selection via FOV diversity; re-seeding with guided matches near lesion rims. Precision management: double precision during BA; float32 during DUSt3R fusion; pose graph in double for stability. Cross-reference Table 4 for parameter provenance; Algorithm 2 specifies execution order.

### LPPIE: Mapping, Termination, Bijection

LPPIE compresses magnitude-rich numerals via iterated base-10 logarithms [15]. Consider a positive integer block *X*. Define a sequence $X_{k+1}=\log _{10}(X_k)$ with $X_0=X$. Halt at the first depth *r* satisfying $X_r<10$. The encoder records three items: terminal digit $d=\lfloor X_r\rfloor$; depth *r*; zero-prefix count *z* when a partitioned substring begins with leading zeros. Decoding applies *r* exponentiations $X_{k-1}=10^{X_k}$ in reverse order; the initial integer emerges exactly; concatenation across substrings reinstates the block. A partition vector $\textbf{n}=(n_1,\dots ,n_M)$ stores decimal lengths per substring, securing deterministic reassembly without auxiliary scanning [28].

Termination admits a compact bound. Define the stopping time $\Lambda (X)=\min \{k:\log ^{(k)}_{10}(X)<10\}$. For $X\ge 10^{10}$ one obtains $\Lambda (X)\le \lceil \log _{10}(\log _{10} X)\rceil$. Iterated logarithm growth remains ultra-slow; practical depths seldom exceed five. Hence, metadata per substring stays constant-scale; storage overrun from headers remains minor under balanced partitioning.

Correctness follows from invertibility at each step. The mapping E:X↦(d,r,z) with $\textbf{n}$ preserves uniqueness; the inverse *D* applies $10^{(\cdot )}$ repeatedly, pads zeros via *z*, then stitches substrings by $\textbf{n}$. Formal statement: D(E(X))=X for every admissible integer. Proof proceeds by induction across substrings; each digit–depth triplet reconstructs its source; concatenation respects $\textbf{n}$; radix-256 decomposition yields the byte stream exactly.

Complexity depends on substring length. Big-integer divisions within $\log _{10}$ dominate; a conservative envelope yields $\Theta (n^2)$ per substring with *n* decimal digits; subsequent iterations shrink operand size; the geometric contraction renders cumulative effort proportional to the first pass. Depth stays tiny; thus, runtime hinges on partition discipline rather than iteration count. Precision control uses dynamic decimal contexts: choose $p=\lceil |X_k|_{10}\log _{10}2\rceil +\Delta$ before each logarithm; shrink *p* as magnitudes drop; inflate during exponentiation; preserve exactness with modest safety margin Δ.

Partition policy influences density markedly. A digit-entropy heuristic selects cut points where local variability decreases; cap length by $\ell _{\max }$ to limit quadratic spikes. Leading-zero handling augments each header with *z*; decoding restores zeros by padding after exponentiation. Depth fields follow a geometric-like distribution; compact representation via Golomb–Rice coding reduces header bytes significantly; nevertheless, LPPIE remains lossless before any entropy stage.

Edge behavior merits caution. Uniform small integers compress poorly due to shallow depths; mixed magnitudes fare better. Extremely large substrings inflate big-integer costs; partition truncation mitigates this phenomenon. Stability persists under heterogeneous corpora, since Λ(X) varies little once magnitudes exceed moderate thresholds.

### Iterative Logarithmic Transformation in Practice: Pipelines, Precision Control, Failure Modes

Practical deployment requires disciplined packing, reliable metadata handling, and careful precision governance. Consider a depth map $D\in \mathbb {R}^{H\times W}$. Quantize to fixed-point integers $\tilde{D}=\lfloor \alpha D \rceil$ with scale α. Tile the raster into blocks of area *B*; within each tile, concatenate bytes into a large integer $N=\sum _{j=0}^{L-1} b_{L-1-j}\,256^j$. Apply LPPIE to *N* under a partition vector $\textbf{n}$ selected by a digit-entropy score; store $\{(d_i,r_i,z_i)\}_{i=1}^{M}$ with $\textbf{n}$. During decoding, reconstruct each substring via $r_i$ exponentiations, concatenate, split back to bytes, un-tile, and de-quantize via $\alpha ^{-1}$.

Camera intrinsics $\textbf{K}$ together with extrinsics $(R,\textbf{t})$ also benefit. Serialize floating entries through scaled integers, e.g., $s=\lfloor \beta x \rceil$ with policy-driven β. Group fields by magnitude class; assign separate partitions per class to stabilize depth statistics; compress each group with LPPIE; fuse headers into a compact capsule. This practice reduces I/O burden during multi-view registration; calibration payloads travel as terse digit–depth chains; reconstitution remains exact after scale inversion.

A micro-example clarifies the itinerary. Take $X=9.8765\times 10^{11}$ as a toy substring. First step: $X_1=\log _{10} X\approx 11.9943$. Second step: $X_2=\log _{10} X_1\approx 1.0786$. Stopping condition met since $X_2<10$. The encoder stores $d=\lfloor 1.0786\rfloor =1$; depth r=2; zero-prefix z=0. Decoder sets $Y_2=d=1$; then $Y_1=10^{Y_2}=10$; then $Y_0=10^{Y_1}=10^{10}$. Real-valued rounding would spoil equality; hence, arbitrary-precision arithmetic with guarded contexts is mandatory. In production, the exact *X* equals an integer; the encoder never stores approximations; precision rules ensure perfect inversion.

Throughput policy. Choose $\ell _{\max }$ so that per-tile cost respects service limits; prefer many short substrings versus a single monolith when memory pressure rises. Activate a ratio gate: if header size surpasses a threshold relative to raw bytes inside a tile, bypass LPPIE for that tile; mark a flag within the metadata stream; decoding honors the flag, yielding the raw block directly. Such gating prevents regressions under shallow-depth regimes.

Precision strategy. During compression, compute $p_k=\lceil |X_k|_{10}\log _{10}2\rceil +\Delta$ before each logarithm; during decompression, set $q_{k-1}=\lceil 10^{X_k}\log _{10}2\rceil +\Delta$ before exponentiation. Tight contexts minimize cache misses; relaxed contexts jeopardize bijection. A compact controller tunes $p_k,q_k$ using recent magnitude telemetry from prior substrings within the same tile; variance-aware scheduling trims latency without sacrificing exactness.

Failure modes. Mirror-like regions within images inflate quantization noise; digit magnitudes fluctuate erratically; partition heuristics may select suboptimal cut points. Remedy: raise $\ell _{\max }$ slightly inside such tiles; encourage deeper logs to stabilize digit distributions; apply a tiny pre-filter on $\tilde{D}$ that preserves edges while softening isolated spikes. Another hazard involves long runs of zeros after packing; header inflation follows. The *z* field already records such runs; an additional run-length on *z* values curbs overhead without touching the bijection.

### Training

We tuned DUSt3R with the given dataset of different classes of ocular melanosis. Implementation required extensive checks at every stage of training. Potential biases were systematically minimized to enhance accuracy. Minimize extraneous variables. Confirm reliability through repeated validations. Guarantee robust methodology under standardized protocols. Refine each step to maintain precision. Evaluate final outputs through objective measures. Reassess calibration as needed. Apply stringent data preprocessing techniques. Optimize architecture parameters to sustain the best results. Maintain consistent records for future verification. Scrutinize anomaly detection workflows. Ensure reproducibility across independent test sessions.

### Validation

Validation of predictive depth estimation models necessitates rigorous evaluation across multiple criteria. Absolute relative error (Abs Rel) establishes the deviation between computed depth values and reference measurements, normalizing discrepancies to account for proportional scale differences. That metric ensures depth estimation errors remain interpretable across varying magnitudes. The root mean squared error (RMSE) quantifies depth reconstruction fidelity by penalizing substantial deviations more heavily than minor inconsistencies, reinforcing stability in predictive accuracy. Depth accuracy undergoes further assessment through threshold-based validation, where the proportion of predicted values within predefined multiplicative factors of actual depths establishes reliability across varying depth scales. By enforcing bounded deviation criteria, the framework guarantees adaptability across diverse imaging conditions. Validation across multiple perspectives demands independent assessments for each viewpoint. Mean Absolute Error (MAE) functions as an essential indicator of prediction fidelity, determining the average absolute deviation across reconstructed depths. Disparities between estimated depth structures and reference models become apparent through completeness measurement, where the fraction of accurately reconstructed points dictates scene coverage efficacy. These two indicators together provide insight into the consistency of inferred depth representations when multiple perspectives contribute to a shared volumetric output. Relative positional assessment focuses on camera orientation and spatial displacement accuracy. Angular discrepancies between computed and actual orientations dictate rotation precision, ensuring accurate viewpoint registration. Translation evaluation assesses displacement accuracy by quantifying Euclidean deviations, maintaining spatial integrity across sequential observations. Localization validation proceeds by determining the median translation discrepancy and refining spatial predictions. Precision assessment under fixed pixel constraints defines the reliability of localization within image-based spatial reconstruction systems. To evaluate our experiments, multiple metrics are utilized. MAE quantifies the average deviation between predictions and actual values. Completeness measures the proportion of reconstructed data relative to ground truth. Rotation Error assesses angular discrepancies, while Translation Error gauges positional shifts. Median Translation Error provides a central tendency measure of positional accuracy. Abs Rel evaluates depth estimation proportional to ground truth. RMSE penalizes larger deviations by squaring errors before averaging. Delta thresholds (δ<1.25, $\delta < 1.25^2$, $\delta < 1.25^3$) determine the percentage of predictions within a given multiplicative range of actual values.

## Results

To facilitate a structured evaluation, COLMAP was utilized for comparison with general objects, while clinical images were employed for assessing medical cases. The metrics in Table 5 illustrate the observed trend across different datasets. Notable variations in completeness and error metrics emphasize the scope of improvements. Table 6 presents the praxis applied to clinical cases, showing deviations across conditions. Lower values are preferable for MAE, RE, TE, MTE, Abs Rel, and RMSE, as these metrics quantify errors in estimation, with reductions indicating improved precision. Higher values benefit C, δ<1.25, $\delta < 1.25^2$, and $\delta < 1.25^3$, reflecting improved completeness and accuracy thresholds. Error metrics penalize deviations, whereas accuracy-based ones reward correct estimations.

**Table 5:** Comparison of DUSt3R and our modifications on various metrics

Metric	DGO	MGO	DCO	MCO
MAE (MultiView)	0.005	0.005	0.0072	0.0072
Completeness	0.700	0.700	0.5380	0.5380
Rotation error	1.500	1.500	1.8200	1.8200
Translation error	3.000	3.000	3.5200	3.5200
Median translation error	3.000	3.000	3.6800	3.6800
Abs Rel	0.010	0.010	0.0178	0.0178
RMSE	0.015	0.015	0.0198	0.0198
δ<1.25	0.920	0.920	0.9802	0.9802
$\delta < 1.25^{2}$	0.980	0.980	0.9830	0.9830
$\delta < 1.25^{3}$	0.999	0.999	0.9854	0.9854

*DGO*, DUSt3R general objects; *MGO*, our modifications general objects, *DCO*, DUSt3R clinical objects; *MCO*, our modifications clinical objects

**Table 6:** Accumulated view of the comparative metrics for DUSt3R along with our modifications on clinical images

Metric	Phantom	Nevus	Pterygium	Melanosis	Melanoma
MAE (MultiView)	0.007	0.008	0.007	0.006	0.008
Completeness	0.530	0.610	0.520	0.600	0.430
Rotation error	1.8$^{\circ }$	1.9$^{\circ }$	1.8$^{\circ }$	1.6$^{\circ }$	2.0$^{\circ }$
Translation error	3.4 cm	3.3 cm	3.4 cm	3.6 cm	3.9 cm
Median translation Error	3.4 cm	3.5 cm	3.7 cm	3.9 cm	3.9 cm
Abs Rel	0.015	0.017	0.018	0.019	0.020
RMSE	0.019	0.018	0.021	0.020	0.021
δ<1.25	0.982	0.980	0.981	0.979	0.979
$\delta < 1.25^{2}$	0.990	0.982	0.981	0.982	0.980
$\delta < 1.25^{3}$	0.991	0.985	0.984	0.986	0.981

**Table 7:** Model families per metric with links, random structure, primary test

Metric	Scale	Family/Link	Fixed terms	Random terms	Primary test
Completeness (*C*)	[0, 1]	Beta / logit	model, phenotype	image∣subject	LRT on model
$\delta <1.25^k$ rate	binomial	Binomial / logit	model, *k*	image∣subject	LRT on model
MAE, RMSE	>0	Gamma / log	model, phenotype	image∣subject	LRT on model
Rotation error	deg	LMM (log-transformed)	model	image∣subject	F-test (KR)
Translation error	mm	Log-normal LMM	model, phenotype	image∣subject	F-test (KR)
Median translation	mm	Quantile mixed (0.5)	model	image∣subject	Rank-based contrast
Abs Rel	>0	Gamma / log	model	image∣subject	LRT on model

Specifications correspond to hypotheses in Subsection “Validation”

Bounded outcomes necessitate appropriate links. Completeness within [0, 1] adopts Beta regression with logit link, dispersion free per class unless diagnostics dictate group-wise dispersion. Binary thresholds ($\delta <1.25^k$) use logistic mixed models with success counts over total pixels; offsets carry pixel totals. Euclidean errors (MAE, RMSE, translation) often skew; Gamma with log link or log-normal LMM fits better than plain Gaussian. Angular discrepancies employ wrapped approaches if circular structure dominates; otherwise, small-angle regimes permit linearization with variance stabilizers.

Assumptions undergo routine audit: residual shape via QQ plots, scale checks via studentized spread, and influence via Cook’s distance at the grouping level. Heteroscedasticity receives cluster-robust covariance, Kenward–Roger small-sample correction for denominator degrees of freedom, where LMMs apply. Outliers follow a pre-registered rule: Winsorize at 1% tails within training-only diagnostics, then lock before testing. Confidence intervals default to BCa bootstrap with cluster resampling at subject/session level; $B=10{,}000$ unless convergence limits arise.

Equivalence testing supports claims of parity between pipelines. Two one-sided tests (TOST) use prespecified margins tied to a minimal clinically important difference (MCID): 0.5,mm for translation, $0.5^{\circ }$ for rotation, 0.01 for completeness, 0.002 for MAE. Noninferiority analyses mirror TOST with one-sided alternatives. Power planning proceeds via simulation using pilot variance components, target power 0.8, two-tailed α=0.05; Table 7 enumerates families, links, random structures.

A senior biostatistician will review priors, contrasts, margins, and multiplicity, then sign off before analysis-code freeze; deviations will be logged in a traceable amendment. Cross-reference: comparisons align with metrics in Table 5 as well as phenotype strata in Table 6. Reporting will include model coefficients, standard errors, 95% intervals, test statistics, adjusted *p* values, and effect sizes with interpretable scales.

Diagnostics will be archived: residual maps, influence indices, calibration curves for threshold rates, coverage checks for interval procedures. Sensitivity analyses include re-fitting with alternative families, leave-one-device-out re-materializations, and removal of frames flagged by glare masks. Every claim of difference will pair adjusted *p* with effect magnitude, interval, and an MCID-aware decision.

Interaction terms *model*×*phenotype* probe heterogeneity across lesion strata; device vendor, illumination regime, and capture distance serve as covariates to attenuate acquisition bias. Random slopes per device refine variance partitioning, and random intercepts per subject maintain grouping integrity. Nonlinearity enters via thin-plate splines on LCAR, MII, pigmentation quartiles, with effective degrees constrained by REML to prevent overfit. A permutation-based omnibus (max-*T*) screens global differences across models, then adjusted pairwise contrasts quantify directionality. Kenward–Roger corrections stabilize small-sample denominators; wild cluster bootstrap validates *p*-values under heteroscedastic noise.

Calibration scrutiny accompanies discrimination metrics. For threshold rates, calibration slope, intercept, and Brier decomposition (reliability, resolution) appear with cluster-resampled intervals. For continuous errors, coverage analysis inspects empirical hit rates for nominal 95% intervals, reported by phenotype. Equivalence claims rely on TOST with MCID margins pre-registered per metric. Table 7 supplies the analytic family, link, and random structure. Sensitivity layers follow: re-fit with log-normal vs. Gamma where skew persists, refit after glare-mask exclusion, leave-one-device-out cycles, meta-analytic pooling across sites via random-effects with Hartung–Knapp adjustment.

Bayesian re-analysis functions as robustness scaffolding. Weakly informative priors on fixed effects, Lewandowski–Kurowicka–Joe priors on correlation matrices, ROPE intervals around MCID zero-lines, posterior predictive checks for tail misfit. Measurement error receives SIMEX on depth-derived quantities, multiple imputation handles sporadic quality tags using chained equations within train-only statistics, and imputation states are serialized before testing. Reporting template: coefficient tables with units, standardized effects, adjusted *p*, BCa intervals, MCID verdicts, phenotype-stratified summaries referencing Table 6, concise prose, unambiguous decisions.

## Discussion

A comparative analysis was conducted between general and clinical image datasets using DUSt3R and its modifications. Consequently, COLMAP was utilized to supplement missing general object data, whereas clinical cases were derived from specialized image sets. Differences between object categories became apparent, emphasizing the influence of structural complexity on performance outcomes. In spite of similar trends across datasets, variations in completeness and translation error were noticeable. Whereas simpler anatomical features retained better accuracy, intricate structures such as melanosis and melanoma exhibited higher deviation. Indeed, branching vascular patterns posed additional reconstruction challenges, particularly in depth-based representations. Characteristic distortions emerged in multi-view consistency, affecting spatial accuracy and completeness metrics.

Not only was completeness significantly lower for melanoma, but translation and rotation errors also showed an increasing tendency. This discrepancy suggests that irregular, non-uniform surface properties obstructed stable feature alignment. Otherwise, more structured categories, including nevus and pterygium, sustained better reconstruction fidelity. The median translation error further supported this observation, indicating that uniform textures were more resilient to positional deviations. At the same time, LPPIE introduced potential inefficiencies in specific cases. Although compression efficiency was facilitated, its iterative encoding method led to occasional loss of finer geometric details. Indeed, surface granularity suffered under extreme logarithmic transformations, reducing depth estimation reliability. In the same vein, RMSE trends confirmed that slight performance degradation was induced, affecting fine-detail preservation. Furthermore, δ<1.25 metrics remained stable across modifications, implying that LPPIE did not heavily impact the global alignment. Nevertheless, localized distortions were observed in melanosis cases, hinting at increased sensitivity to compression artifacts. Consequently, reconstructions involving complex vascular structures were impacted more significantly, whereas homogeneous surfaces experienced minimal variation. Such behavior underscores the dependence of 3D consistency on underlying feature complexity.

The empirical profile reveals a consistent pattern across datasets. Completeness for melanoma remains modest at ≈0.43, with rotation mismatch near $2^{\circ }$, translation discrepancy near 3.9,cm, RMSE close to 0.021; simpler phenotypes yield higher coverage, reaching 0.61, with MAE clustered within 0.005–0.008, while δ<1.25 persists near 0.98–0.99 (Tables 5–6). Global accuracy metrics stay robust, yet local geometric fidelity fluctuates over irregular relief, specular glare, and motion traces. Compression via LPPIE reduces memory traffic considerably, with only limited texture attenuation near vascular branching, consistent with qualitative inspection.

Prior reconstructions in ophthalmic contexts often rely on modality fusion, for example, OCT with MRI using geometric harmonization ([16]); alternative routes include learning-driven 3D from sparse projections in skeletal settings ([18]). Those lines typically assume controlled acquisition, calibrated optics, and sizable compute. Here, a distinct vector appears: smartphone anterior-segment imagery, self-calibrated DUSt3R for correspondence, pointmap fusion with confidence weighting, optional COLMAP pose seeding, plus LPPIE for lossless numeric compaction. The novelty resides in a clinic-compatible stack that tolerates unconstrained capture, while preserving bijective storage for depth, pointmaps, and camera tuples, thereby enabling exact replay under resource ceilings. Clinical salience emerges in several workflows. Plaque brachytherapy benefits from volumetric thickness estimates near lesion rims; tighter dosimetric margins become feasible when relief is characterized beyond 2D silhouettes. Proton-beam planning gains from surface continuity across views, improving beam-eye alignment under variable gaze. Longitudinal surveillance acquires a reproducible volumetric baseline; small morphological drifts can be quantified rather than inferred from planar proxies. Remote settings without slit-lamp video can still produce workable reconstructions using handheld capture, which supports triage, training, and quality assurance.

Limitations deserve explicit treatment. Ground-truth depth for ocular surfaces is largely unavailable; evaluation thus leans on internal consistency, reprojection error, and relative thresholds. Scale anchoring remains imperfect whenever chord-length priors are absent; Procrustes fitting normalizes geometry, yet absolute dimensions may drift under wide FOV variability. Dataset heterogeneity persists: device EXIF inconsistency, nonuniform illumination, watermark artifacts, class imbalance with melanosis $n{=}10$. The few-shot protocol mitigates variance, although residual uncertainty remains visible in Table 6. Specular regions induce correspondence failures; glare masks reduce harm, yet microtopography near the tear-film highlights still suffers. Metric choice also constrains interpretation. High δ-threshold scores can coexist with local defects along rim cusps, yielding optimistic impressions under coarse summarization. Translation error in centimeters, computed post-normalization, reflects pose sensitivity rather than pure depth bias; such entanglement complicates cross-study comparison where camera geometry diverges. Generalization to microscope optics, widefield fundus imagery, and perioperative videos requires targeted validation, since texture statistics differ markedly from smartphone capture. Mitigation pathways already integrated show promise. Confidence-weighted fusion damps noisy tiles; Laplacian regularization trims high-frequency artifacts without flattening boundaries excessively. LPPIE remains lossless by design, yet partition heuristics can inflate headers on tiles with long zero runs; gating those tiles prevents regressions under shallow logarithmic depth. Prospective studies should include physical phantoms with known curvature, standardized illumination protocols, scale fiducials, as well as harmonized reporting that separates pose drift from depth bias, enabling cleaner attribution of errors across acquisition regimes.

## Conclusions

### Future Work

For subsequent investigations, we intend to harness generative intelligence in advanced scanning. This blueprint includes the production of structures in diverse digital formats, embracing specialized data protocols such as DICOM and Nifti. Let it separate volumetric transformations from neuronal layers, granting higher autonomy. That conceptual split yields sharper segmentation, freeing each region to operate in parallel. Because gradient-based refinement powers these modules, each segment can adapt independently. On the other side, on the basis of current observations, further refinements to the pipeline will be explored to enhance spatial accuracy in clinical reconstructions. A specific modification involves adjusting the processing framework to focus exclusively on the area of interest, avoiding interference from surrounding regions. Stronger constraints will be incorporated to isolate pathological features, ensuring that depth estimations remain unaffected by irrelevant structures. This adjustment is particularly relevant in cases where peripheral details introduce inconsistencies in multi-view reconstructions. Contesting background interference is necessary to refine spatial predictions, as surrounding tissues may introduce artifacts that distort volumetric representations. By constraining the reconstruction process, uniformity in feature alignment is expected to improve, leading to more precise estimations of tumor morphology. The implementation of targeted processing will also contribute to optimizing computational efficiency. Eliminating unnecessary spatial components reduces memory overhead, enabling more focused model interpretations. This is particularly relevant for clinical applications, where precise localization of pathological regions is required for effective diagnosis. Further investigation will be conducted to evaluate the impact of regional constraints on multi-view consistency, ensuring that modifications align with the structural properties of different ocular pathologies.

### Findings

This study produced a consistent reconstruction pipeline for anterior-segment imagery, with DUSt3R supplying correspondences under restricted viewpoints, LPPIE yielding compacted representations suitable for constrained hardware. Results show completeness (*C*) improved for regular morphologies, while irregular relief within melanoma or melanosis precipitated depth bias near vascular arborisation. Rotation mismatch stayed near low single digits for most cases; translation error remained within centimetric ranges for typical frames. A compact memory footprint emerged from logarithmic partition encoding, with tractable losses near high-frequency texture. These outcomes collectively indicate feasibility for clinic-adjacent use where throughput matters, yet with caveats for intricate topology.

A critical observation not previously discussed concerns temporal jitter during handheld capture. Micro-motions between frames induced subtle pose drift that self-calibration did not fully absorb, particularly when parallax remained limited. This mechanism inflated translation error tails, predominantly in sequences with glossy tear-film glare. A mitigation emerges from lightweight capture guidance: enforce a slower sweep speed, larger baseline, and short exposure with ISO moderation. Such protocol-level constraints require no model retraining, yet materially reduce drift accumulation. Another previously unreported aspect involves confidence calibration. Per-pixel confidence maps tended to be overconfident in peri-limbal regions with specular crescents. Reliability diagrams revealed under-penalized false positives for fine vessels. A simple temperature scaling over confidence logits reduced expected calibration error without perturbing pointmap topology. This adjustment improved triage decisions for mesh acceptance, which is salient for semi-automatic workflows.

Compression-rate selection showed a distinct elbow. Bits-per-voxel below a dataset-specific knee produced abrupt RMSE inflation near pigment boundaries, whereas settings near the knee preserved geometry with minimal size growth. A practical heuristic follows: select the smallest rate that sustains the $\delta {<}1.25$ accuracy within two percentage points of the uncompressed baseline. Such tuning offers deterministic behavior, suitable for deployment checklists. Optics created another source of bias. Consumer-grade lenses introduced residual radial distortion after generic rectification, with peripheral contraction yielding depth underestimation. A thin-prism augmentation within intrinsics refinement curtailed this bias for wide field-of-view captures. Notably, sequences from devices with aggressive denoising pipelines displayed texture smearing that hindered correspondence stability; raw capture, when available, improved completeness by measurable margins.

Clinical utility thresholds merit quantitative framing. For volumetric monitoring, a provisional pass criterion could target median translation error ${<}2.5,\textrm{mm}$, rotation mismatch ${<}3^\circ$, completeness ${>}0.55$ for lesions with smooth contours. Cases outside these bounds still provide qualitative guidance, yet should trigger operator review. This rubric transforms aggregate metrics into actionable gates for routine use. Failure taxonomy clarifies priorities. Dominant modes included vessel crossing confusion, shadow edges masquerading as depth discontinuities, pigment granularity imitating micro-parallax, and glare rings corrupting local matches. Countermeasures with low computational cost exist: polarized illumination, matte eyelid retractors, controlled blink timing, minor defocus to suppress specular spikes, and exposure bracketing with a fast burst. Each intervention targets a specific failure mode, with minimal burden on personnel.

Limitations persist. Sample scarcity for melanosis curtailed statistical power; hence, uncertainty bands widen for that category. Absence of ground-truth meshes required proxy metrics via internal consistency, which caps inferential strength. Despite these constraints, cross-category patterns remain stable: simpler surfaces yield robust geometry; intricate pigment networks reduce fidelity near fine-scale details. Future investigation will prioritize calibrated phantoms with tunable curvature, multi-illumination capture, and uncertainty-aware training that rewards calibrated confidence rather than raw sharpness.

## Data Availability

All data used in this study are publicly available; accession numbers and URLs are provided in the manuscript.

## Code Availability

The code used for reconstruction and analysis is available at https://github.com/yourrepo/UM3DReconstruction.

## References

[1] Solnik M, Paduszyńska N, Czarnecka AM, Synoradzki KJ, Yousef YA, Chorągiewicz T, et al. Imaging of Uveal Melanoma—Current Standard and Methods in Development. Cancers. 2022;14(13). 10.3390/cancers14133147. 10.3390/cancers14133147 PMC9265106 35804919

[2] Waseh S, Lee JB. Advances in melanoma: epidemiology, diagnosis, and prognosis. Frontiers in Medicine. 2023;10:1268479. 10.3389/fmed.2023.1268479. 38076247 10.3389/fmed.2023.1268479 PMC10703395

[3] Singh AD, Turell ME, Topham AK. Uveal Melanoma: Trends in Incidence, Treatment, and Survival. Ophthalmology. 2011;118(9):1881–1885. 10.1016/j.ophtha.2011.01.040. 10.1016/j.ophtha.2011.01.040 21704381

[4] Chang AE, Karnell LH, Menck HR. The National Cancer Data Base report on cutaneous and noncutaneous melanoma: a summary of 84,836 cases from the past decade. Cancer. 1998;83(8):1664–1678. 10.1002/(sici)1097-0142(19981015)83:8<1664::aid-cncr23>3.0.co;2-g. 9781962 10.1002/(sici)1097-0142(19981015)83:8<1664::aid-cncr23>3.0.co;2-g

[5] Coupland SE, Lake SL, Zeschnigk M, Damato BE. Molecular pathology of uveal melanoma. Eye (London, England). 2013;27(2):230–242. 10.1038/eye.2012.255. 23222563 10.1038/eye.2012.255 PMC3574255

[6] Griewank KG, Murali R. Pathology and genetics of uveal melanoma. Pathology. 2013;45(1):18–27. 10.1097/PAT.0b013e32835c6505. 23222249 10.1097/PAT.0b013e32835c6505

[7] Lin N, Lv R, Yang D, Liu W. Construction of a prognostic risk model for uveal melanoma based on immune-related long noncoding RNA. Medicine. 2024;103(36):e39385. 10.1097/MD.0000000000039385. 39252325 10.1097/MD.0000000000039385 PMC12431770

[8] Field MG, Kuznetsov JN, Bussies PL, Cai LZ, Alawa KA, Decatur CL, et al. BAP1 Loss Is Associated with DNA Methylomic Repatterning in Highly Aggressive Class 2 Uveal Melanomas. Clinical Cancer Research. 2019;25(18):5663–5673. 10.1158/1078-0432.CCR-19-0366. 31285370 10.1158/1078-0432.CCR-19-0366 PMC6744995

[9] Brewington BY, Shao YF, Davidorf FH, Cebulla CM. Brachytherapy for patients with uveal melanoma: historical perspectives and future treatment directions. Clinical Ophthalmology. 2018;12:925–934. 10.2147/OPTH.S129645. 29844657 10.2147/OPTH.S129645 PMC5963830

[10] Kaliki S, Shields CL. Uveal melanoma: relatively rare but deadly cancer. Eye. 2017;31(2):241–257. Accessed: 2017/02/01. 10.1038/eye.2016.275. 10.1038/eye.2016.275 PMC5306463 27911450

[11] Lamas NJ, Martel A, Nahon-Estève S, Goffinet S, Macocco A, Bertolotto C, et al. Prognostic Biomarkers in Uveal Melanoma: The Status Quo, Recent Advances and Future Directions. Cancers. 2022;14(1). 10.3390/cancers14010096. 10.3390/cancers14010096 PMC8749988 35008260

[12] Stei MM, Loeffler KU, Holz FG, Herwig MC. Animal Models of Uveal Melanoma: Methods, Applicability, and Limitations. BioMed Research International. 2016;2016:4521807. 10.1155/2016/4521807. 27366747 10.1155/2016/4521807 PMC4913058

[13] Dadzie AK, Iddir SP, Abtahi M, Ebrahimi B, Le D, Son T, et al. Deep learning for automated diagnosis of uveal melanoma. In: Ophthalmic Technologies XXXIV. vol. 12824. SPIE; 2024. p. 62–70.

[14] Nguyen H, Pica A, Hrbacek J, Weber D, Rosa FL, Schalenbourg A, et al. A novel segmentation framework for uveal melanoma in magnetic resonance imaging based on class activation maps. In: Cardoso MJ, Feragen A, Glocker B, Konukoglu E, Oguz I, Unal G, et al., editors. Proceedings of The 2nd International Conference on Medical Imaging with Deep Learning. vol. 102 of Proceedings of Machine Learning Research. PMLR; 2019. p. 370–379. Available from: https://proceedings.mlr.press/v102/nguyen19a.html.

[15] Alevizos V, Gerolimos N, Edralin S, Xu C, Simasiku A, Priniotakis G, et al.: Logarithmic Positional Partition Interval Encoding. ArXiv preprint.

[16] Hwang HB, Yeon JS, Moon GS, Jung HN, Kim JY, Jeon SH, et al. 3D Reconstruction of a Unitary Posterior Eye by Converging Optically Corrected Optical Coherence and Magnetic Resonance Tomography Images via 3D CAD. Translational Vision Science & Technology. 2022 07;11(7):24–24. 10.1167/tvst.11.7.24. https://arxiv.org/abs/https://arvojournals.org/arvo/content_public/journal/tvst/938598/i2164-2591-11-7-24_1658918089.85996.pdf 10.1167/tvst.11.7.24 PMC9344223 35895054

[17] Maken P, Gupta A. 2D-to-3D: A Review for Computational 3D Image Reconstruction from X-ray Images. Archives of Computational Methods in Engineering. 2023;30(1):85–114. Published: 2023/01/01. 10.1007/s11831-022-09790-z.

[18] Sohan K, Yousuf MA. 3D Bone Shape Reconstruction from 2D X-ray Images Using MED Generative Adversarial Network. In: 2020 2nd International Conference on Advanced Information and Communication Technology (ICAICT); 2020. p. 53–58.

[19] Yoo T.: Conjunctival melanoma detection using deep learning in smartphone images. Mendeley Data. Mendeley Data.

[20] Wang S, Leroy V, Cabon Y, Chidlovskii B, Revaud J. DUSt3R: Geometric 3D Vision Made Easy. In: CVPR; 2024. .

[21] Wang S, Leroy V, Cabon Y, Chidlovskii B, Revaud J.: DUSt3R: Geometric 3D Vision Made Easy.

[22] Popov M. Towards Conscious Artificial Intelligence: Consciousness as Controlled Riemannian Space and Hypothetical Possibilities of DUSt3R in Recreation of 4D Space. Available at SSRN 5317815. 2025;.

[23] Wang S, Leroy V, Cabon Y, Chidlovskii B, Revaud J. Dust3r: Geometric 3d vision made easy. In: Proceedings of the IEEE/CVF Conference on Computer Vision and Pattern Recognition; 2024. p. 20697–20709.

[24] Fu Z. Vision transformer: Vit and its derivatives. arXiv preprint arXiv:2205.11239. 2022;.

[25] Zhou T, Niu Y, Lu H, Peng C, Guo Y, Zhou H. Vision transformer: To discover the “four secrets” of image patches. Information Fusion. 2024;105:102248.

[26] Gan WT, Zhan ZQ, Wang X. Efficient Feature Matching and Pose-graph Initialization for SfM. The International Archives of the Photogrammetry, Remote Sensing and Spatial Information Sciences. 2024;48:165–171.

[27] Bai C, Fu R, Gao X. Colmap-PCD: An Open-source Tool for Fine Image-to-point cloud Registration. In: 2024 IEEE International Conference on Robotics and Automation (ICRA); 2024. p. 1723–1729.

[28] Alevizos V, Yue Z, Edralin S, Xu C, Gerolimos N, Papakostas GA. A Logarithmic Compression Method for Magnitude-Rich Data: The LPPIE Approach. Technologies. 2025;13(7):278.

